# Physical Activity Patterns of the Spanish Population Are Mostly Determined by Sex and Age: Findings in the ANIBES Study

**DOI:** 10.1371/journal.pone.0149969

**Published:** 2016-02-25

**Authors:** Juan Mielgo-Ayuso, Raquel Aparicio-Ugarriza, Adrián Castillo, Emma Ruiz, José Manuel Ávila, Javier Aranceta-Batrina, Ángel Gil, Rosa M. Ortega, Lluis Serra-Majem, Gregorio Varela-Moreiras, Marcela González-Gross

**Affiliations:** 1 ImFINE Research Group, Department of Health and Human Performance, Technical University of Madrid, Madrid, Spain; 2 Spanish Nutrition Foundation (FEN), Madrid, Spain; 3 Department of Preventive Medicine and Public Health, University of Navarra, Pamplona, Spain; 4 CIBEROBN (Physiopathology of Obesity and Nutrition CB12/03/30038), Instituto de Salud Carlos III (ISCIII), Madrid, Spain; 5 Department of Biochemistry and Molecular Biology II, and Institute of Nutrition and Food Sciences, University of Granada, Granada, Spain; 6 Department of Nutrition, Faculty of Pharmacy, Complutense University of Madrid, Madrid, Spain; 7 Research Institute of Biomedical and Health Sciences, University of Las Palmas de Gran Canaria, Department of Health Sciences, Las Palmas de Gran Canaria, Spain; 8 Department of Pharmaceutical and Health Sciences, Faculty of Pharmacy, CEU San Pablo University, Madrid, Spain; Institute for Health & the Environment, UNITED STATES

## Abstract

**Background:**

Representative data for the Spanish population regarding physical activity (PA) behaviors are scarce and seldom comparable due to methodological inconsistencies.

**Aim:**

Our objectives were to describe the PA behavior by means of the standardized self-reported International Physical Activity Questionnaire (IPAQ) and to know the proportion of the Spanish population meeting and not meeting international PA recommendations.

**Material and Methods:**

PA was assessed using the IPAQ in a representative sample of 2285 individuals (males, 50.4%) aged 9–75 years and living in municipalities of at least 2,000 inhabitants. Data were analyzed according to: age groups 9–12, 13–17, 18–64, and 65–75 years; sex; geographical distribution; locality size and educational levels.

**Results:**

Mean total PA was 868.8±660.9 min/wk, mean vigorous PA 146.4±254.1 min/wk, and mean moderate PA 398.1±408.0 min/wk, showing significant differences between sexes (p<0.05). Children performed higher moderate-vigorous PA than adolescents and seniors (p<0.05), and adults than adolescents and seniors (p<0.05). Compared to recommendations, 36.2% of adults performed <150 min/week of moderate PA, 65.4% <75 min/week of vigorous PA and 27.0% did not perform any PA at all, presenting significant differences between sexes (p<0.05). A total of 55.4% of children and adolescents performed less than 420 min/week of MVPA, being higher in the later (62.6%) than in the former (48.4%). Highest non-compliance was observed in adolescent females (86.5%).

**Conclusion:**

Sex and age are the main influencing factors on PA in the Spanish population. Males engage in more vigorous and light PA overall, whereas females perform more moderate PA. PA behavior differs between age groups and no clear lineal increase with age could be observed. Twenty-seven percent of adults and 55.4% of children and adolescents do not meet international PA recommendations. Identified target groups should be addressed to increase PA in the Spanish population.

## Introduction

Regular physical activity (PA) contributes to the primary and secondary prevention of several chronic diseases and is associated with a reduced risk of premature all-cause death in all age groups [1,2]. Likewise, regular PA prevents unhealthy body mass gain and obesity, whereas sedentary behaviors may promote them [3].

Given that PA is a complex and multidimensional behavior, precise and reliable quantification can be difficult [4]. Although subjective methods have their limitations [5], the International Physical Activity questionnaire (IPAQ) has since become the most widely used physical activity questionnaire for every age stage [6], suggesting that characteristics of age, sex, locality size, and social status should be considered when designing PA strategies [7].

According to several international and national organizations, to promote and maintain health all adults over 18 years of age should perform moderate-intensity aerobic PA for a minimum of 150 minutes, or vigorous-intensity aerobic PA for a minimum of 75 minutes, every week of the year [8–11]. Combinations of moderate- and vigorous-intensity PA (MVPA) can be performed to meet this recommendation. Along the same lines, children and young people aged 5–17 years should accumulate at least 60 minutes of MVPA daily [10] throughout the year.

Scientific studies determining the PA patterns in Spain are scarce, despite the positive impact of PA on public health [12–14]. Available data from different sources, have given discrepant results regarding the Spanish population. Eurobarometer data from 2014 indicate that Spaniards who exercised or participated in sports at least once a week had increased 6% with respect to Eurobarometer data from 2010 (44% vs. 38%) [15,16]. The Spanish National Health Survey (2011/2012) revealed that when considering both primary and leisure-time PA activities, 40.9% of adults (49.4% males, 32.4% females) aged 15–69 years perform vigorous to moderate PA weekly using IPAQ [17]. However, this study did not split the sample into different age groups, especially into adults and adolescents. Moreover, they did not study the PA behaviors of senior people (> 69 ages). In a representative study in Catalonia, 37.9% and 38.8% reported engaging in vigorous and moderate levels of PA, respectively. Also, the prevalence of activity during leisure time was 16.1% [18]. Nevertheless, all these data lack comparability because different physical activity questionnaires, definitions, and criteria for adequate levels of PA were used.

On the other hand, there have been important social and lifestyle changes in the Mediterranean area in the last years which could have contributed to make important changes in leisure time activities which have notably contributed in reducing PA [19]. Given there's sound evidence showing that PA is associated with a range of health benefits, to address properly public health policies, it is necessary to have a clear picture of the population's PA behavior.

Within this context, the main objective of the present study was to describe the PA activity behavior by means of an established standardized instrument for the assessments of self-reported PA behavior (IPAQ), and the distribution by sex, age group, geographical distribution, and locality size, in a national representative sample of the Spanish population. The second objective was to know the proportion of the Spanish population meeting and not meeting international PA recommendations. The present findings from the ANIBES (“Anthropometry, Intake, and Energy Balance in Spain”) study provide an overall PA picture and can be used to define target public health policies based on scientific evidence in the near future.

## Materials and Methods

The overall design, protocol, and methodology of the ANIBES study have previously been reported in detail [20,21].

### Study participants

The design of the ANIBES study aimed to define a sample size that is representative of all individuals living in Spain (excluding the autonomous cities of Melilla and Ceuta in North Africa) aged 9–75 years and living in municipalities of at least 2,000 inhabitants. The sample for the ANIBES Study was designed based on 2012 census data published by the INE (Instituto Nacional de Estadística/Spanish Bureau of Statistics) for Sex, Age, Habitat Size and Region. The total sample size was calculated based on a 0.05 probability of Type I error (rejecting a null hypothesis when it is true) and 0.1 probability of Type II error (accepting a null hypothesis when it is wrong) in the main outcome of the study (energy intake). The initial potential sample was 2,634 individuals, and the final sample comprised 2009 individuals (2.23% error and 95.5% confidence interval). In addition, a boost sample was recruited for the youngest age groups (9–12; 13–17), and oldest age group (65–75 years) so as to include at least 200 individuals per age group (error +/- 6.9%). For this analysis, the final sample plus boost consisted of 213 children aged 9–12 years, 211 adolescents aged 13–17 years, and 206 seniors. Therefore, the random sample plus booster was 2,285 participants. However, the booster data are only analysed in the context of the analysis of these specific subgroups and not in the context of the analysis of the main random sample. The ANIBES sample reflected the distribution in the population living in Spain (1013 males, 50.4%, and 996 females, 49.6%).

For the sampling, the following variables were taken into account: age groups ((children (9–12 years), adolescents (13–17 years), adults (18–64 years), and seniors (65–75 years)); adults group ((young adults (18–30 years), middle adults (31–49 years) and old adults (50–64 years)), sex; geographical distribution (Northeast, Levant, South, West, North-Central, Barcelona, Madrid, and Balearic and Canary Islands); and locality size (2,000–30,000 inhabitants, rural population; 30,000–200,000 inhabitants, semi-urban population; and over 200,000 inhabitants, urban population). Geographical distributions were grouped into 4 different regions (Center, Atlantic, Mediterranean and South). Additionally, educational level was considered for sample adjustment.

Several exclusion criteria were applied: individuals living in an institutional setting (e.g. colleges, nursing homes, hospitals); individuals following a therapeutic diet due to recent surgery or any medical prescription; potential participants with any transitory illness (e.g. flu, gastroenteritis, chicken pox) at the time fieldwork was undertaken; and individuals employed in areas related to consumer science, marketing or the media. However, individuals under the following conditions were considered eligible for inclusion: those following dietary protocols, such as for the prevention of hypertension, diabetes, hypercholesterolemia, or hyperuricemia; pregnant and lactating women; people with diagnosed allergies and/or food intolerance; or those with metabolic disease, for example, hyperthyroidism or hypothyroidism.

The ANIBES study was conducted using stratified multistage sampling, and 128 sampling points were considered, for more coverage and representativeness. No pre-recruitment was considered, to minimize the risk of bias in responses.

### Fieldwork

All participants were informed of the protocol and risks/benefits and all adults signed a written consent form prior to participation. In the same line, informed written consent from children and adolescents was obtained from participants and parents or guardians. The final protocol was approved by the Ethical Committee for Clinical Research of the Region of Madrid, Spain. The study was coded as “FEN 2013”, and approved on May 31, 2013. Fieldwork for the ANIBES study was from mid-September 2013 to mid-November 2013 (3 months).

### Physical activity level

During a face-to-face visit, trained researchers administered the IPAQ [22,23] to adults; the modified IPAQ, according to the HELENA study [24,25], was administered to children and adolescents. This IPAQ was not specifically validated for children under the age of 12. Children answered the questionnaires, with the parents being present.

Data collected from the IPAQ surveys were summed within each PA domain to estimate the total time spent in PA related to occupational, transportation, household, and leisure activities. The questionnaire was scored using established methods, available on the IPAQ website (www.ipaq.ki.se). Minutes at each intensity level exceeding 180 per day were truncated to 180 to avoid extreme outliers. However, in MVPA the maximum was 360 minutes/day (2520 minutes/week), as a sum of moderate PA (180 min/day) and vigorous PA (180 min/day). Likewise, in total PA the maximum was 540 minutes/day (3780 minutes/week), as a sum of light PA (180 min/day), moderate PA (180 min/day) and vigorous PA (180 min/day). These data were summarized to report PA by categories, according to the measured PA (light, moderate, vigorous, MVPA and total PA).

### Statistical treatment

Data are presented as means, standard deviation, median, percentiles, ranges and percentages. Analyses were performed using SPSS version 22.0 (SPSS, Inc, Chicago, Illinois, USA). The level of significance was set at 5%. Once all the information on PA was transformed into min/wk, the Kolmogorov-Smirnoff test was used to test if the variables followed a normal distribution, to decide between parametric or non-parametric analysis. As variables were non-normally distributed, non-parametric tests were performed. Differences by age, geographical distribution, locality size, and educational level and between sexes were performed using Kruskal-Wallis for independent samples (K-samples). To determine if differences existed between different points into sample quotas a post-hoc test (pairwise comparisons controlling significance) was applied. Likewise, for differences between sexes Mann-Whitney test was used (2 samples).

On the other hand, participants were classified according to whether they did meet or not meet each of the international PA recommendations for their age group. This distribution was performed according to different age groups and sexes. A Z-test was used to discern the differences between two proportions among different sex and age groups.

## Results

Table 1 describes the sample distribution divided by age, sex, locality size, geographical distribution, and educational levels. Tables 2–5 present descriptive PA data of the total sample and divided by age, sex, locality size, geographical distribution, and educational levels. Tables 6 and 7 show the proportions of participants who did not meet international PA recommendations. In the annex, Table 8 presents descriptive data of light PA which will not be further commented.

**Table 1:** Distribution of total sample, and total and booster sample, in each group of the study.

	Total (n)	Males (n)	Females (n)
Age groups			
9–12 years*	213	126	87
13–17 Years*	211	137	74
18–64 Years	1655	797	858
65–75 Years*	206	99	107
Adult groups			
18–30 years	422	210	212
31–49 years	807	389	418
50–64 years	426	198	228
Geographical distribution			
Center	455	240	215
Atlantic	314	157	157
Mediterranean	704	353	351
South	536	262	274
Locality size			
Rural	682	344	338
Semi-urban	683	358	325
Urban	644	310	334
Educational levels			
Primaries	744	381	363
Secondary	859	432	427
University	406	199	207

* Total plus booster sample.

**Table 2:** Total physical activity (min/wk).

	Total Sample	Males	Females
	Mean±SD	Mean±SD	Mean±SD
	Median (range)	Median (range)	Median (range)
Total	868.8±660.9	857.4±679.0	880.4±637.2
	740(0–3780)	700(0–3780)	785(0–3780)
Age Groups			
a. 9–12 years*	1179.0±911.7	1330.3±990.0	960.4±733.3
	935(0–3780)	1060(120–3780)	720(135–3780)
b. 13–17 years*	936.8±762.5^a^	1066.6±811.6^a^	694.4±597.0^a^
	740(90–3780)	845(105–3780)	505(90–3150)
c. 18–64 years*	856.6±637.7^a^	824.9±645.0^a^	886.1±608.4^b^
	735(0–3360)	690(0–3360)	820(0–3360)
d. 65–75 years	806.5±616.3^a^	824.8±627.3^a^^,^^b^	870.3±637.1
	720(0–2655)	660(30–2520)	780(0–2655)
Adult Groups			
e. 18–30 years	764.0±597.2	788.5±605.1	739.5±589.7
	615(0–2970)	620(0–2940)	600(0–2970)
f. 31–49 years	897.8±650.1^e^	870.0±652.6	923.5±647.4^e^
	840(0–3360)	735(0–3360)	842.5(0–3360)
g. 50–64 years*	870.2±645.0^e^	776.1±667.9	953.5±613.5^e^
	775(0–3240)	585(0–3240)	900(0–2940)
Geographical Distribution			
h. Center	836.9±660.1	836.6±693.7	837.1±623.8
	720(0–3780)	695(0–3780)	720(0–3240)
i. Atlantic	967.1±677.4^h^	928.8±679.5	1005.5±675.4
	840(0–3215)	795(0–3215)	930(0–2820)
j. Mediterranean	880.9±637.1	897.8±730.6	869.9±564.8
	762.5(0–3780)	720(0–3780)	840(0–2880)
k. South	822.1±674.6^i^	786.7±631.7	855.2±711.7^i^
	690(0–3780)	630(0–3780)	750(0–3780)
Locality Size			
l. Rural	860.7±663.4	857.0±6756.4	863.0±651.5
	750(0–3780)	720(0–3780)	790(0–3780)
m. Semi-urban*	898.8±690.7	862.5±717.6	934.4±659.1
	750(0–3780)	690(0–3780)	840(0–3780)
n. Urban	847.8±635.9	851.0±715.0	844.5±605.7
	727.5(0–3150)	690(0–2940)	762.5(0–3150)
Educational Levels			
o. Primaries	924.5±676.5	949.1±719.8	889.7±629.8
	840(0–3780)	840(0–3780)	840(0–3780)
p. Secondary*	855.6±663.2	818.2±675.8^o^	893.0±650.3
	700(0–3780)	620(0–3780)	785(0–3240)
q. University	794.8±613.1^o^	766.0±588.8^o^	822.4±640.2
	630(0–2880)	630(0–2640)	650(0–2880)

*: *p* < 0·05 indicates statistical significance between sexes (Mann-Whitney for independent samples test).

Significant differences among different points inside the groups (*p* < 0·05) by Bonferroni or Games-Howell post-hoc test after check differences by Kruskal-Wallis for independent samples test.

^a^: vs 9–12 years.

^b^: vs 13–17 Years.

^e^: vs 18–30 years.

^h^: vs Center.

^i^: vs Atlantic.

^o^: vs Primaries.

**Table 3:** Vigorous physical activity (min/wk).

	Total Sample	Males	Females
	Mean±SD	Mean±SD	Mean±SD
	Median (range)	Median (range)	Median (range)
Total*	146.4±254.1	197.7±283.3	93.4±209.0
	0.0 (0.0–1260.0)	60.0(0.0–1260.0)	0.0(0.0–1260.0)
Age Groups			
a. 9–12 years*	273.2±309.6	344.4±315.7	171.2±272.6
	180.0(0.0–1260)	270.0(0.0–1260.0)	75.0(0.0–1200.0)
b. 13–17 years*	222.2±290.0	275.2±297.7^a^	125.5±249.0
	120.0 (0.0–1260.0)	180.0(0.0–1260.0)	5.5(0.0–1200.0)
c. 18–64 years*	149.2±264.1^a^	210.6±301.7^a^^,^^b^	92.2±209.1^a^
	0.0(0.0–1260.0)	40.0(0.0–1260.0)	0.0(0.0–1260.0)
d. 65–75 years	38.3±107.8^a^^,^^b^^,^^c^	41.9±112.2^a^^,^^b^^,^^c^	35.0±104.0^a^^,^^b^^,^^c^
	0.0 (0.0–600.0)	0.0(0.0–600.0)	0.0(0.0–600.0)
Adult Groups			
e. 18–30 years*	181.7±275.2	280.1±318.7	84.3±177.1
	7.5 (0.0–1260)	180.0(0.0–1260. 0)	0.0(0.0–1080)
f. 31–49 years*	156.4±269.3	206.9±295.3^e^	109.5±234.8
	0.0(0.0–1260.0)	60.0(0.0–1260.0)	0.0(0.0–1260.0)
g. 50–64 years*	103.3±235.9^e^^,^^f^	144.2±280.3^e^^,^^f^	67.7±181.7^f^
	0.0(0.0–1260.0)	0.0(0.0–1260.0)	0.0(0.0–1260.0)
Geographical Distribution			
h. Center*	145.8±256.2	190.1±287.0	96.4±206.4
	0.0(0.0–1260.0)	0.0(0.0–1260.0)	0.0(0.0–1260.0)
i. Atlantic*	153.2±269.3	188.4±277.1	118.0±257.4
	0.0(0.0–1260.0)	30.0(0.0–1260.0)	0.0(0.0–1260.0)
j. Mediterranean*	139.6±239.4	195.1±272.4	82.4±184.5
	0.0(0.0–1260.0)	60.0(0.0–1260.0)	0.0(0.0–1080.0)
k. South*	151.0±264.3	212.2±299.3	92.6±211.3
	0.0(0.0–1260.0)	80.0(0.0+-1260.0)	0.0-(0.0–1260.0)
Locality Size			
l. Rural *	157.5±270.1	220.4±305.7	92.0±209.0
	0.0(0.0–1260.0)	80.0(0.0–1620.0)	0.0(0.0–1260.0)
m. Semi-urban *	141.1±252.7	175.4±262.2	103.4±236.5
	0.0(0.0–1260.0)	0.0(0.0–1260.0)	0.0(0.0–1260.0)
n. Urban *	139.3±240.1	196.2±280.6	86.5±180.1
	0.0(0.0–1260.0)	60.0(0.0–1260.0)	0.0(0.0–1200.0)
Educational Levels			
o. Primaries*	142.2±264.0	208.3±310.5	73.6±182.1
	0.0(0.0–1260)	0.0(0.0–1260.0)	0.0(0.0–1260.0)
p. Secondary*	146.6±249.1	194.5±272.6	97.3±211.7
	0.0(0.0–1260)	60.0(0.0–1260.0)	0.0(0.0–1260.0)
q. University*	152.1±249.9	182.8±252.5	122.5±244.3^o^
	0.0(0.0–1260.0)	60.0(0.0–1260.0)	0.0(0.0–1260.0)

*: *p* < 0·05 indicates statistical significance between sexes (Mann-Whitney for independent samples test).

Significant differences among different points inside the groups (*p* < 0·05) by Bonferroni or Games-Howell post-hoc test after check differences by Kruskal-Wallis for independent samples test.

^a^: vs 9–12 years.

^b^: vs 13–17 years.

^c^: vs 18–64 years.

^e^: vs 18–30 years.

^f^: vs 31–49 years.

^o^: vs Primaries.

**Table 4:** Moderate physical activity (min/wk).

	Total Sample	Males	Females
	Mean±SD	Mean±SD	Mean±SD
	Median (range)	Median (range)	Median (range)
Total*	398.1±408.4	310.2±363.1	487.6±432.9
	240.0 (0.0–1260)	180.0(0.0–1260.0)	360.0(0.0–1260)
Age Groups			
a. 9–12 years*	343.4±362.4	381.9±371.6	288.1±342.4
	180.0(0.0–1260.0)	285.0 (0.0–1260.0)	165.0 (0.0–1260.0)
b. 13–17 years*	233.0±306.6	273.5±335.1^a^	157.3±251.7
	105.0 (0.0–1260.0)	125.0 (0.0–1260.0)	75.0(0.0–1260.0)
c. 18–64 years*	415.2±411.9^a^^,^^b^	312.9±361.5^a^^,^^b^	510.3±432.7^a^^,^^b^
	270.0(0.0–1260.0)	180.0 (0.0–1260.0)	420.0(0.0–1260.0)
d. 65–75 years*	390.1±435.3^b^	315.7±404.3^a^	458.9±453.2^b^
	210.0(0.0–1260.0)-	120.0(0.0–1260.0)	300.0 (0.0–1260.0)
Adult Groups			
e. 18–30 years*	316.3±354.5	246.3±299.0	385.3±390.4
	180.0(0.0–1260.0)	155.0 (0.0–1260.0)	240.0(0.0–1260.0)
f. 31–49 years*	439.6±418.6^e^	329.9±376.5^e^	541.8±430.8^e^
	300.0 (0.0–1260.0)	180.0(0.0–1260.0)	420.0(0.0–1260.0)
g. 50–64 years*	467.0±435.7^e^	350.1±385.0^e^	568.5±452.2^e^
	300.0(0.0–1260.0)	199.5(0.0–1260.0)	480.0(0.0–1260.0)
Geographical Distribution			
h. Center*	347.8±378.8	275.1±342.2	428.4±400.7
	210.0(0.0–1260.0)	127.5(0.0–1260.0)	360.0(0.0–1260.0)
i. Atlantic*	443.9±428.8^h^	352.7±363.8^h^	533.1±469.1
	270.0(0.0–1260.0)	240.0(0.0–1260.0)	360.0(0.0–1260.0)
j. Mediterranean*	412.7±407.2	323.0±372.8	502.4±421.2
	240.0(0.0–1260.0)	184.5(0.0–1260.0)	420.0(0.0–1260.0)
k. South*	396.8±419.8	298.1±367.0	489.4±445.5
	240.0(0.0–1260.0)	137.5(0.0–1260.0)	360.0(0.0–1260.0)
Locality Size			
l. Rural *	407.0±417.2	312.8±377.6	503.4±435.5
	240.0(0.0–1260.0)	180.0(0.0–1260.0)	420.0(0.0–1260.0)
m. Semi-urban*	415.2±422.1	326.7±376.0	514.4±447.2
	240.0(0.0–1260.0)	180.0(0.0–1260.0)	420.0(0.0–1260.0)
n. Urban *	370.6±382.1	288.6±329.7	445.7±411.6
	240.0(0.0–1260.0)	165.0(0.0–1260.0)	315.0(0.0–1260.0)
Educational Levels			
o. Primaries*	427.9±434.6	334.8±382.4	523.5±463.6
	240.0(0.0–1260.0)	180.0(0.-1260.0)	420.0(0.0–1260.0)
p. Secondary*	401.5±404.2	302.2±357.5	502.2±424.6
	240.0(0.0–1260.0)	180.0(0.0–1260.0)	360.0(0.0–1260.0)
q. University*	338.2±360.9^o^^,^^p^	278.3±336.1	395.7±375.1^o^^,^^p^
	210.0(0.0–1260.0)	180.0(0.-1260.0)	280.0(0.0–1260.0)

*: *p* < 0·05 indicates statistical significance between sexes (Mann-Whitney for independent samples test).

Significant differences among different points inside the groups (*p* < 0·05) by Bonferroni or Games-Howell post-hoc test after check differences by Kruskal-Wallis for independent samples test.

^a^: vs 9–12 years.

^b^: vs 13–17 years.

^e^: vs 18–30 years.

^h^: vs Center

^o^: vs Primaries.

^p^: vs Secondary.

**Table 5:** Moderate-vigorous physical activity (min/wk).

	Total Sample	Males	Females
	Mean±SD	Mean±SD	Mean±SD
	Median (range)	Median (range)	Median (range)
Total*	543.7±507.0	507.8±507.9	580.1±505.5
	420(0–2520)	360(0–2520)	420(0–2520)
Age Groups			
a. 9–12 years*	616.6±578.6	724.4±600.4	459.3±509.2
	435(0–2520)	532.5(0–252)	262.5(0–2460)
b. 13–17 years*	452.2±494.9^a^	545.6±503.1^a^	279.8±430.0
	270(0–2520)	420(0–2520)	147.5(0–2460)
c. 18–64 years*	564.4±509.3^b^	523.5±515.5^a^	602.5±500.8^b^
	420(0–2520)	360(0–2520)	480(0–2520)
d. 65–75 years	428.4±462.4^a^^,^^c^	357.6±425.8^a^^,^^b^^,^^c^	493.8±486.6^b^^,^^c^
	240(0–1680)	200(0–1680)	360(0–1620)
Adult Groups			
e. 18–30 years	498.0±462.8	526.4±467.4	469.9±457.6
	390(0–2340)	420(0–1995)	360(0–2340)
f. 31–49 years*	596.1±520.8^e^	536.8±531.0	651.3±505.5^e^
	450(0–2520)	390(0–2520)	540(0–2520)
g. 50–64 years*	570.3±526.0	494.4±534.2	636.2±510.8^e^
	420(0–2520)	300(0–2520)	600(0–2520)
Geographical Distribution			
h. Center	493.6±480.3	465.5±477.1	525.9±481.9
	375(0–2460)	360(0–1995)	400(0–2460)
i. Atlantic	593.6±546.1	540.3±533.3	646.9±556.4
	405(0–2520)	360(0–2520)	427.5(0–2520)
j. Mediterranean*	551.3±494.1	518.1±515.4	584.7±469.6
	420(0–2520)	360(0–2520)	510(0–2340)
k. South	547.9±521.9	511.2±507.8	581.5±532.0
	420(0–5160)	360(0–2520)	427.5(0–2520)
Locality Size			
l. Rural	564.3±518.0	533.7±522.0	595.2±512.7
	420(0–2520)	405(0–2520)	450(0–2520)
m. Semi-urban*	555.9±526.4	501.41±513.3	615.3±534.8
	420(0–2520)	360(0–2520)	450(0–2520)
n. Urban	509.9±473.1	484.5±482.0	532.5±464.9
	375(0–2520)	340(0–2100)	420(0–2460)
Educational Levels			
o. Primaries	568.4±523.0	543.8±526.0	595.7±520.0
	420(0–2520)	420(0–2520)	450(0–2520)
p. Secondary*	547.7±506.7	497.6±507.4	598.3±501.4
	420(0–2520)	360(0–2520)	450(0–2460)
q. University	490.2±476.4	461.2±467.0	518.2±484.8
	360(0–2520)	300(0–2100)	360(0–2520)

*: *p* < 0·05 indicates statistical significance between sexes (Mann-Whitney for independent samples test).

Significant differences among different points inside the groups (*p* < 0·05) by Bonferroni or Games-Howell post-hoc test after check differences by Kruskal-Wallis for independent samples test.

^a^: vs 9–12 years.

^b^: vs 13–17 years.

^c^: vs 18–64 years.

^e^: vs 18–30 years.

**Table 6:** Percentage of sample who did not meet PA international recommendations by sex and age groups.

	%	ICC (95%)	%	ICC (95%)	%	ICC (95%)
**Adults**						
	**Total (n = 1861)**		**Males (n = 897)**		**Females (n = 964)**	
	< 75 min/week VPA					
Total*	65.4	63.2–67.6	56.2	52.9–59.4	74.0	71.2–76.7
a. 18–30*	54.5	49.7–59.3	37.9	31.3–44.5	71.1	64.9–77.3
b. 31–49*	61.1	57.7–64.5	51.7^a^	46.7–56.7	69.8	65.4–74.2
c. 50–64*	74.6^a^^,^^b^	70.5–78.8	70.0^a^^,^^b^	63.6–76.4	78.8	73.4–84.1
d. 65–75	85.4 ^a^^,^^b^^,^^c^	80.6–90.3	84.8^a^^,^^b^^,^^c^	77.7–92.0	86.0^a^^,^^b^	79.3–92.7
	< 150 min/week MPA					
Total*	36.2	34.0–38.4	45.6	42.3–45.6	27.5	24.7–30.3
a. 18–30*	42.2	37.4–46.9	48.8	42.0–55.6	35.5	29.0–42.1
b. 31–49*	33.6^a^	30.3–36.8	45.0	40.0–49.9	23.1^a^	19.0–27.1
c. 50–64*	32.4^a^	27.9–36.9	40.5	33.6–47.4	25.2	19.5–30.9
d. 65–75*	42.2	35.4–49.0	51.5	41.5–61.5	33.6	24.5–42.7
	< 150 min/week MPA or < 75 min/week VPA					
Total*	27.0	25.0–29.0	31.1	28.1–34.1	23.2	20.6–23.2
a. 18–30	26.3	22.1–30.5	22.7	17.0–28.5	29.9	23.6–36.1
b. 31–49*	23.8	20.8–26.7	31.0	26.4–35.6	17.1^a^	13.5–20.8
c. 50–64*	27.9	23.7–32.2	32.5	26.0–39.0	23.9	18.3–29.5
d. 65–75*	39.3 ^a^^,^^b^^,^^c^	32.6–46.0	46.5^a^	36.5–56.5	32.7^b^^,^^c^	23.7–41.7
**Children and adolescents**						
	**Total (n = 424)**		**Males (n = 263)**		**Females (n = 161)**	
	< 420 min/week MVPA					
Total*	55.4	50.7–60.2	44.5	38.4–50.5	73.3	66.4–80.2
e. 9–12*	48.4	41.6–55.1	38.9	30.3–47.5	62.1	51.7–72.5
f. 13–17*	62.6^e^	56.0–69.1	49.6^e^	41.2–58.1	86.5^e^	78.5–94.5

*P<0.05 between sexes (Z-test).

P<0.05 between age groups (Z-test):

^a^: vs 18–30.

^b^: vs 31–49.

^c^: vs 50–64.

^e^: vs 9–12

**Table 7:** Percentage of participants who did not any PA (0 min/week) by sex and age groups.

	%	ICC (95%)	%	ICC (95%)	%	ICC (95%)
Adults						
	Total (n = 1861)		Males (n = 897)		Females (n = 964)	
	Total PA					
Total	3.1	2.4–3.8	2.4	1.5–3.3	3.7	2.6–4.8
a.9-12	0	-	0	-	0	-
b.13-17	0	-	0	-	0	-
c. 18–30	2.4	0.9–3.8	1.4	0–3.0	3.3	0.9–5.8
d. 31–49	4.1	2.7–5.5	3.9	1.9–5.8	4.3	2.3–6.2
e. 50–64	4.2	2.3–6.1	5.0	2.0–8.0	3.5	1.1–6.0
f. 65–75	4.4	1.6–7.2	0		8.4	3.1–13.8
	Vigorous PA					
Total*	54.3	52.2–56.3	43.5	40.7–46.4	65.3	62.5–68.1
a.9-12*	20.2	14.8–25.6	9.5	4.3–14.7	35.6	25.4–45.9
b.13-17*	30.8	24.5–37.1	20.4	13.6–27.3	50.0	38.3–61.7
c. 18–30*	50.0^a^^,^^b^	45.2–54.8	34.6^a^	28.1–41.1	65.4^a^	58.9–71.9
d. 31–49*	55.6^a^^,^^b^	52.2–59.1	46.5^a^^,^^b^	41.5–51.5	64.0^a^	59.4–68.7
e. 50–64	71.1^a^^,^^b^^,^^c^^,^^d^	66.8–75.4	67.0^a^^,^^b^^,^^c^^,^^d^	60.4–73.6	74.8^a^^,^^b^	69.1–80.5
f. 65–75	82.0^a^^,^^b^^,^^c^^,^^d^^,^^e^	76.8–87.3	78.8^a^^,^^b^^,^^c^^,^^d^	70.6–87.0	85.0^a^^,^^b^^,^^c^^,^^d^	78.2–91.9
	Moderate PA					
Total*	21.1	19.5–22.8	25.3	22.8–27.9	16.8	14.6–19.0
a.9-12	15.5	10.6–20.4	12.7	6.8–18.6	19.5	11.0–28.0
b.13-17	25.6	19.7–31.5	24.1	16.8–31.3	28.4^a^	17.9–38.9
c. 18–30*	23.5	19.4–27.5	28.0^a^	21.9–34.1	19.0^b^	13.6–24.3
d. 31–49*	19.8	17.1–22.6	27.4^a^	22.9–31.9	12.9^b^	9.6–16.1
e. 50–64*	17.6	14.0–21.2	23.5	17.6–29.4	12.4^b^	8.1–16.7
f. 65–75	30.1^a^^,^^d^^,^^e^	23.8–36.4	33.3^a^	23.9–42.8	27.1^c^^,^^d^	18.5–35.7
	MVPA					
Total	14.8	13.4–16.3	16.2	14.1–18.3	13.4	11.4–15.4
a.9-12*	4.7	1.8–7.6	1.6	0–3.8	9.2	3.0–15.4
b.13-17	9.0	5.1–12.9	8.0	3.4–12.6	10.8	3.6–18.1
c. 18–30	15.6^a^	12.2–19.1	3.9^a^	1.9–5.8	18.0	12.8–23.2
d. 31–49*	14.6^a^	12.2–17.1	19.6^a^^,^^b^^,^^c^	15.7–23.6	10.0	7.1–12.9
e. 50–64*	16.0^a^	12.5–19.5	20.5^a^^,^^b^^,^^c^	14.9–26.1	11.9	7.7–16.2
f. 65–75	28.2^a^^,^^b^^,^^c^^,^^d^^,^^e^	22.0–34.3	30.3^a^^,^^b^^,^^c^^,^^d^^,^^e^	21.1–39.5	26.2^a^^,^^d^^,^^e^	17.7–34.6
	Light PA					
Total	10.6	9.4–11.9	10.4	8.7–12.2	10.8	9.0–12.7
a.9-12	5.2	2.2–8.2	5.6	1.5–9.6	4.6	0.1–9.1
b.13-17	3.3	0.9–5.8	3.6	0.5–6.8	2.7	0–6.5
c. 18–30	10.9^a^	7.9–13.9	10.9	6.7–15.1	10.9	6.7–15.1
d. 31–49	13.3^a^^,^^b^	10.9–15.6	13.7^b^	10.3–17.1	12.9	9.6–16.1
e. 50–64	12.2^b^	9.1–15.3	15.5^b^	10.4–20.6	9.3	5.5–13.1
f. 65–75*	9.7^d^	5.6–13.8	2.0^d^^,^^e^	0–4.8	16.8^b^	9.6–24.0

*P<0.05 between sexes (Z-test).

P<0.05 between age groups (Z-test):

^a^: vs 9–12.

^b^: vs 13–17.

^c^: vs 18–30.

^d^: vs 31–49.

^e^: vs 50–64.

**Table 8:** Light physical activity (min/wk).

	Total Sample	Males	Females
	Mean±SD	Mean±SD	Mean±SD
	Median (range)	Median (range)	Median (range)
Total	310.3±314.9	329.0±335.9	291.3±290.5
	210.0(0.0–1260.0)	210.0(0.0–1260.0)	210.0(0.0–1260.0)
Age Groups			
a. 9–12 years	396.7±336.0	418.2±367.8	365.6±287.8
	300.0(0.0–1260.0)	305.0(0.0–1260.0)	275.0(0.0–1260.0)
b. 13–17 years	359.0±323.6	377.0±346.4	326.5±301.6
	270.0(0.0–1260.0)	275.0(0.0–2260.0)	227.5(0.0–1260.0)
c. 18–64 years	292.2±307.0^a^^,^	303.8±329.5^a^^,^^b^	28143±284.4^a^
	210.0(0.0–1260.0)	210.0(0.0–1260.0)	210.0(0.0–1260.0)
d. 65–75 years*	378.2±343.6^c^	467.3±350.2^c^	295.7±317.4^a^
	290.0(0.0–1260.0)	420.0(0.0–1260.0)	210.0(0.0–1260.0)
Adult Groups			
e. 18–30 years	266.0±286.3	262.8±293.7	269.1±279.9
	180.0(0.0–1260.0)	180.0(0.0–1260.0)	190.0(0.0–1260.0)
f. 31–49 years	301.8±321.5	334.5±357.4^e^	271.3±280.0
	210.0(0.0–1260.0)	210.0(0.0–1260.0)	210.0(0.0–1260.0)
g. 50–64 years	300.0±298.0	286.7±302.7	311.5±294.5
	210.0(0.0–1260.0)	210.0(0.0–1260.0)	210.0(0.0–1260.0)
Geographical Distribution			
h. Center	327.3±327.3	348.1±351.0	304.2±297.8
	210.0(0.0–1260.0)	210.0(0.0–1260.0)	210.0(0.0–1260.0)
i. Atlantic	362.5±311.2	380.9±332.2	345.3±289.4
	250.0(0.0–1260.0)	260.0(0.0–1260.0)	240.0(0.0–1260.0)
j. Mediterranean	311.8±309.5	345.2±344.9	278.1±365.3^i^
	210.0(0.0–1260.0)	210.0(0.0–1260.0)	210.0(0.0–1260.0)
k. South	264.7±306.7^h^^,^^i^	361.6±300.3^h^^,^^i^^,^^j^	266.8±312.6^i^
	155(0.0–1260.0)	177.5(0.0–1260.0)	150.0(0.0–1260.0)
Locality Size			
l. Rural	281.2±302.5	302.4±330.2	259.5±269.0
	200.0(0.0–1260.0)	202.5(0.0–1260)	180.0(0.0–1260.0)
m. Semi-urban	324.5±322.2^l^	335.4±341.0	311.3±300.6^l^
	210.0(0.0–1260.0)	210.0(0.0–1260.0)	210.0(0.0–1260.0)
n. Urban	326.9±317.3^l^	352.6±332.4^l^	303.0±300.0
	210.0(0.0–1260.0)	210.0(0.0–1260.0)	210.0(0.0–1260.0)
Educational Levels			
o. Primaries*	323.2±316.6	361.2±348.5	284.8±275.4
	210.0(0.0–1260.0)	240.0(0.0–1260.0)	210.0(0.0–1260.0)
p. Secondary	301.0±309.0	312.2±327.9	289.5±289.9
	210.0(0.0–1260.0)	210.0(0.0–1260.0)	210.0(0.0–1260.0)
q. University	306.0±320.3	304.8±324.5	307.2±316.9
	210.0(0.0–1260.0)	210.0(0.0–1260.0)	210.0(0.0–1260.0)

*: *p* < 0·05 indicates statistical significance between sexes (Mann-Whitney for independent samples test).

Significant differences among different points inside the groups (*p* < 0·05) by Bonferroni or Games-Howell post-hoc test after check differences by Kruskal-Wallis for independent samples test.

^a^: vs 9–12 years.

^b^: vs 13–17 years.

^c^: vs 18–64 years.

^e^: vs 18–30 years.

^h^: vs Center

^i^: vs Atlantic.

^j^: vs Mediterranean.

^l^: vs rural.

### Total PA

In the total sample, children had greater total PA than adolescents, adults and seniors (p< 0.05). Young adults had less total PA than other adults (p< 0.05) (Table 2). In males, children had higher total PA levels than other groups (*p* < 0.05); and adolescents had also higher total PA than seniors (*p* < 0.05). In females, adolescents had lower total PA than girls and adults (p< 0.05). Young adult females engaged in lower total PA than the other two adult groups (p< 0.05).

On the other hand, children and adolescent males practiced more total PA than females (p< 0.05). However, in total adults and old adults females had higher PA than males (p< 0.05). Total sample and females living in the Atlantic region showed higher levels of total PA than those living in the South of Spain (p<0.05). Likewise, females from semi-urban localities and with secondary educational levels s performed more total PA than males in the same group (p< 0.05).

Moreover, in the total sample and in females, participants with primary or lower educational levels presented higher total PA values than those with a university-level education (*p* < 0.05). Likewise, females with primary or lower educational levels presented higher total PA values than those with secondary levels (p< 0.05).

### Vigorous PA

Vigorous PA data are presented in Table 3. Overall, males were more vigorously active than females independently of the classification groups (p< 0.05). In the total sample, seniors (65–75 years) were less vigorously active (p< 0.05) regarding other age groups. Likewise, adults carried out less vigorous PA than children (p< 0.05). On the other hand, young adult males (18–30 years) showed greater vigorous PA than other adults; and middle adults than old adults (p< 0.05). Moreover, older adult females (50–64 years) performed less vigorous PA than middle-aged female adults (p< 0.05).

No differences according to geographical distribution and locality size were observed. However, females with university studies showed higher vigorous PA than those with primary studies (p<0.05).

### Moderate PA

Moderate PA data are shown in Table 4. Females in general were more active than males (p< 0.001), except in children and adolescents. Adolescents engaged in less moderate PA than adults and seniors (*p* < 0.05) and young adults overall were less active than the other adults (p< 0.05). On the other hand, both sexes separately followed the same pattern as the general population (p<0.05).

By geographical distribution, the whole sample and males living in southern Spain performed less moderate PA than those living in the Atlantic region (p< 0.05). The whole sample and females with university-level education had the lowest levels of moderate PA (*p*< 0.05).

### Moderate-vigorous PA

MVPA among the Spanish population is shown in Table 5. Children and adolescent males practiced more MVPA than females (p<0.05). However, in adults, especially in middle and old adults it was the opposite (p<0.05). In the same line, females living in the Mediterranean area and those having secondary studies showed higher MVPA than males (p<0.05).

Regarding age groups, children performed higher MVPA than adolescents and seniors (p<0.05), and adults than adolescents and seniors. Among males, boys presented higher MVPA than the other age groups, contrary than seniors (p< 0.05). Among females, adolescents performed less MVPA than adults and seniors (p< 0.05).

### Adherence to PA recommendations

The percentage of Spaniards who did not comply with the international recommendations is shown in Table 6.

Participants who did not meet the recommendation of almost 75 min/week of vigorous PA were 65.4% in the total sample (74.0% of females and 56.2% of males; p<0.05). Moreover, significant differences between sexes in every age group (p<0.05) were observed, except in seniors. In the total sample and in males, participants who did not meet this recommendation increased with age (p<0.05). However, in females, seniors presented more participants (86.0%) who did not meet the 75 min/week of vigorous PA recommendation than young (71.1%) and middle-aged (69.8%) adults.

Participants who did not meet the recommendation of almost 150 min/week of moderate PA were 36.2% (27.5% of females and 45.6% of males; p<0.05). Likewise, in every age group, males showed more participants who did not meet 150 min/week of moderate PA recommendation than females (p<0.05). In this aspect, the percentage of adult males who did not meet recommendations did not show significant differences among age groups. However, in females, among middle age adults (23.1%) there were less participants who did not meet this recommendation (p<0.05) than among young adults (35.5%).

We also calculated participants who did not meet any of these recommendations (<150 min/week moderate PA or < 75 min/week vigorous PA). They were 27.0% in the total sample (31.1% in males and 23.2% in females; p<0.05). We observed significant different behavior between sexes in middle and old adults, and seniors (p<0.05), having more people who did not meet these recommendations in males than in females. In adult males, the number of participants who did not meet these recommendations (p<0.05) was higher in seniors (46.5%) than in young adults (22.7%). However, in females, only 17.1% of middle-aged adults did not meet these recommendations compared to young adults (29.9%) and seniors (86.6%) (p<0.05). Likewise, seniors presented higher number of subjects who did not meet the recommendations than older adults (23.9%) (p<0.05).

Among children and adolescents, 55.4%, did not meet the international recommendations for these age groups (420 min/week MVPA), being 73.3% in females and 44.5% in males (p<0.05). Likewise, there were no significant differences between sexes and age groups (p>0.05), presenting in both sexes that adolescents had higher rates of non-fulfilling PA guidelines than children.

### 0 minutes/week of PA

Subjects who do not perform any daily vigorous PA increase with increasing age in both sexes (Table 7). Regarding moderate PA, in the total sample, seniors had a higher % of inactive participants, followed by adolescents and young adults (p<0.05). In males, highest inactivity percentages were observed in seniors, followed by young and middle-aged adults (p<0.05). On the contrary, among females, adolescents had the highest inactivity percentage, followed by seniors and girls (p<0.05). Combining MVPA, there is a tendency of increasing percentage of inactive subjects with increasing age group, with the exception of young adult males having lower percentage of inactivity than adolescent males. In females, highest inactivity percentage was observed in the senior group, followed by young adult females (p<0.05). Regarding light PA, highest inactivity percentage was observed in the total sample for middle-aged adults, in males for older adults and in females in seniors (p<0.05).

## Discussion

This study describes PA patterns of a nationally representative sample of the Spanish population aged 9–75 years. To our knowledge, no previous research has reported such PA patterns for the entire Spanish population, showing light, moderate and vigorous PA levels according to different age groups, geographical distribution, locality size, and educational levels. The main findings of this study indicate that although the means of vigorous and/or moderate PA were higher than international recommendations [9–11], a large percentage of participants did not meet these recommendations. In this study, mean values mask the real picture because 65.4% and 36.2% of adult participants had not acceptable values of vigorous (75 min/week) and moderate (150 min/week) PA, respectively. However, when taken together, Spanish adults not meeting any of these target recommendation levels drops to 27.0%. However, results are worse in children and adolescents, as 55.4% did not meet the PA recommendation for this age group (420 min/week of MVPA). Also worthwhile to mention is that in the total Spanish population 54.3% and 21.1% never performs vigorous and moderate PA, respectively.

Several international organizations have attempted to summarize their recommendations regarding the most appropriate amount of PA for healthy body mass, maintaining health, and preventing chronic diseases in the population [9–11]. To improve cardiorespiratory and muscular fitness, bone health, and cardiovascular and metabolic health, and to reduce symptoms of anxiety and depression, children and young people aged 5–17 years should accumulate at least 60 minutes of MVPA daily (420 min/wk). Most daily PA should be comprised of aerobic activities within the context of family, school, and community activities, such as playing games and participating in sports, transportation, recreation, physical education, or planned exercise [10]. These recommendations are met, on average, by Spanish children and adolescents of both sexes. However, 62.1% of female children, 86.5% of female adolescents, 38.9% of male children and 49.6% of male adolescents did not meet these recommendations. Worldwide, 80% of adolescents aged between 13 and 15 years do not achieve this target [26]. This proportion is even higher across the EU-28 in which 83% of 11- to 15-year-olds are estimated to be, for the most part, physically inactive [27].

In the same way, the percentage of compliance with MVPA recommendations for European allies countries children varies considerably between sexes and countries and is generally low, ranging from 15% in Switzerland to 43% in Slovakia among 11-year-olds, from 12% in France to 42% in Slovakia among 13-year-olds, and from 8% in Israel to 37% in Slovakia among 15-year-olds. Overall, boys and younger children were more active than girls and older children [28]. Given that young people have massively incorporated new technologies into their leisure-time activities [29] and that there has been a decreasing trend in Spain of active commuting to school [30], MVPA and active commuting (mainly walking) to school should be encouraged, among other activities [31,32].

Adults aged 18–64 years should perform at least 150 min/wk of moderate-intensity aerobic PA or at least 75 min/wk of vigorous-intensity aerobic PA, or an equivalent combination of MVPA [9–11]. Furthermore, evidence supports between 150 and 250 min/wk of moderate-intensity PA to prevent weight gain effectively, and more than 200 min/wk have been associated with clinically significant weight loss [33]. In the ANIBES study, Spanish adults presented a higher mean than those general recommendations. Nevertheless, examining these results more closely, it can be observed that 45.6% of male and 27.5% of female adults did not meet the recommendation of 150 min/wk of moderate PA. The World Health Organization recommends an additional 150 min/wk of moderate PA for additional health benefits [10]. Similarly, 56.2% of males and 74% of female adults did not meet the recommendations for vigorous PA. Moreover, seniors had the lowest compliance with the recommended 75 min/wk of vigorous-intensity PA, at approximately 85.4% (males: 84.8%; females: 86.0%) of total adults. In the same line 27.0% (males: 31.1%; females: 23.2%) of the total adults population did not meet any of both recommendations. Regarding vigorous-intensity PA recommendations, our data were 6% lower than those of the Spanish National Health Survey, in which 33.6% of the total Spanish population aged 18–69 years (31.3% males and 35.8% females) did not meet PA recommendations [17]. These differences could be due to the different methodology of classifying PA. Eurobarometer 2014 data showed that over a period of 1 week, 54% of European respondents did not engage in any vigorous activity and 44% did not engage in any moderate activity [16]. According to the most recent Center for Disease Control reports, 52% of males and 43% of females meet guidelines for MVPA in the United States [34]. Salavane et al. (2012) found that almost two-thirds of French adults engaged in MVPA; however, 6% reported not engaging in any physical activity at all [35].

Recommendations for adults aged 18–65 and adults aged over 65 years are similar, but this age group is often the least physically active [9–11]. Concretely, we have shown that although the mean of moderate PA was higher than recommendations in both sexes among the Spanish population, 51.5% of male and 33.6% of female seniors did not meet the 150 min/wk of moderate-intensity PA. Guallar-Castillón et al. observed that in Spain, more than 40% of older adults are sedentary [36], which could be due to increased difficulty walking for 1 hour without resting or walking up 10 steps [37]. In a former study, we observed decreasing levels of physical fitness with age [38]. Specifically, lower body strength was already low at the age of 65 [38]. It appears that there is an increase in PA among seniors in the last decade in both the United States [37,39,40] and Spain [41–43]. However, around 85% of senior people of both sexes did not meet the recommended 75 min/wk of vigorous-intensity PA in our study. Taking all these results together, may be separate recommendations should be adopted and implemented for the latter population, which could be more motivating and feasible for them.

In the last years, regional governments in Spain have launched different policies regarding promotion of PA [44]. This could be, among others, the reason why PA levels vary between each study area. Forty percent of people who live in the central or southern part of Spain did not meet the recommendations for moderate PA, whereas only 30% failed to meet these recommendations in the Atlantic and Mediterranean areas. Pardo et al. (2014) found that three-quarters of the Catalan population (Mediterranean region) reached the recommended levels of health-enhancing physical activity [18], whereas 50% of the population were active in Galicia (Atlantic region) [45]; in the Madrid area (Central region) only 27.1% fulfilled the recommendations of the American College of Sports Medicine in another study [46]. However, these differences could be due to the different methodologies and criteria used to define physical activity.

Several published studies have revealed that in most countries, less educated people engage in less PA than people with higher educational levels [47,48]. Despite these data, in the ANIBES study, participants with primary levels of education were more active than more highly educated participants. Interestingly, a recent study performed in Chile agrees with our data [49]. Apart from methodological aspects, these contradictory data could be related to the economic downturn that has affected countries like Spain since 2008, which was still the case when the ANIBES data were obtained.

The ANIBES study has several strengths which include the careful design, protocol, and methodology used, conducted among a random representative sample of the Spanish population aged 9–75 years. The validated questionnaires used to collect information on physical activity have shown good reliability and reproducibility. One limitation of this study is its cross-sectional design, which provides evidence for associations but not causal relationships. Measures of physical activity relied on self-reports and could be biased, although a careful multistep quality control procedure was implemented to minimize bias. Additional limitations could be the high type I error rate due to hundreds of comparisons and secondary outcome (also inflates type I error rate).

In conclusion, the main influencing factors on PA among the Spanish population are sex and age and educational level in females. Males perform more vigorous and light PA at all ages, whereas females perform more moderate PA but only in adulthood. PA behaviour differs between age groups and no clear lineal increase or decrease with age could be observed. Average data show levels of moderate and vigorous PA that comply with international recommendations; however, 27% of the Spanish adults and 55.4% of Spanish children and adolescents do not meet international recommendations of 150 min/week of moderate PA or 75 min/week of vigorous PA and 420 min/week of MVPA, respectively. Considering our results, corrective measures should be taken on identified target groups to increase PA in the Spanish population.
